# Plan Selection, Enrollee Risk, and Health Spending on the Patient Protection and Affordable Care Act Individual Marketplaces, 2019

**DOI:** 10.1001/jamanetworkopen.2023.4529

**Published:** 2023-03-30

**Authors:** Graham Treasure, David M. Anderson, Lauren Hatcher, Alexandra E. Makhoul, Darren Johnson, Jenna Stefan, Kevin N. Griffith

**Affiliations:** 1 Department of Medicine, Vanderbilt University Medical Center, Nashville, Tennessee; 2 Margolis Center for Health Policy, Duke University, Durham, North Carolina; 3 Department of Population Health Sciences, Duke University, Durham, North Carolina; 4 Department of Anesthesia, Hospital of the University of Pennsylvania, Philadelphia; 5 Wakely Consulting Group LLC, an HMA Company, Tampa Bay, Florida; 6 Department of Health Policy, Vanderbilt University Medical Center, Nashville, Tennessee; 7 Partnered Evidence-Based Policy Resource Center, VA Boston Healthcare System, Boston, Massachusetts

## Abstract

**Importance:**

The Patient Protection and Affordable Care Act (ACA) individual marketplaces are a source of insurance for millions of residents in the US. However, the association between enrollee risk, health spending, and metal tier selection remains unclear.

**Objectives:**

To describe individual marketplace enrollees’ metal tier selections by risk score and assess enrollees’ health spending by metal tier, risk score, and spending type.

**Design, Setting, and Participants:**

This retrospective, cross-sectional study analyzed claims data from the Wakely Consulting Group ACA database, a deidentified claims database built on data voluntarily submitted by insurers. Enrollees with continuous, full-year enrollment in on-exchange or off-exchange ACA-qualified health plans during the 2019 contract year were included. Data analysis was conducted from March 2021 to January 2023.

**Main Outcomes and Measures:**

Enrollment totals, total spending, and out-of-pocket cost were calculated, stratified by metal tier and the Department of Health and Human Services (HHS) Hierarchical Condition Category (HCC) risk score for 2019.

**Results:**

Enrollment and claims data were obtained for 1 317 707 enrollees (53.5% female; mean [SD] age, 46.35 [13.43] years) across all census areas, age groups, and sexes. Of these, 34.6% were on plans with cost-sharing reductions (CSRs), 75.5% did not have an assigned HCC, and 84.0% submitted at least 1 claim. Compared with enrollees in bronze plans (17.2%), enrollees were more likely to be classified in the top HHS-HCC risk quartile if they selected platinum (42.0%), gold (34.4%), or silver (29.7%) plans. The highest share of enrollees with $0 total spending was noted with the catastrophic (26.4%) and bronze (22.7%) plans, while gold plans had the lowest share (8.1%). Median total spending was lower among bronze plan enrollees ($593; IQR, $28-$2100) vs platinum ($4111; IQR, $992-$15 821) or gold ($2675; IQR, $728-$9070). Within the top risk score decile, CSR enrollees had less average total spending than any other metal tier by more than 10%.

**Conclusions and Relevance:**

In this cross-sectional study of the ACA individual marketplace, enrollees who selected plans with higher actuarial value also had greater mean HHS-HCC risk scores and health spending. The findings suggest these differences may be associated with variation in benefit generosity by metal tier, enrollee’s perceptions of future health needs, or other barriers to care access.

## Key Points

**Question:**

What are enrollee risk scores and health spending by metal tier (bronze, silver, gold, platinum) in the Patient Protection and Affordable Care Act individual marketplace?

**Findings:**

In this cross-sectional study including 1.3 million enrollees, large variation in health spending and out-of-pocket costs by metal tier were observed. Individual risk was associated with enrollment in plans with higher actuarial value; however, many enrollees in low-value plans were classified as high risk and faced substantial out-of-pocket costs.

**Meaning:**

The findings of this cross-sectional study suggest metal tier is associated with both enrollee risk and future health spending, which may reflect variation in benefit generosity, enrollee’s perceptions of future health needs, or access barriers.

## Teaser

This cross-sectional study evaluates the selection of plans available in the Patient Protection and Affordable Care Act (ACA) individual marketplace by individuals associated with their risk score assigned by the Department of Health and Human Services.

## Introduction

Since their launch in 2014, the Patient Protection and Affordable Care Act (ACA) individual marketplaces have been a source of insurance for millions of individuals in the US each year^1^ and provide enrollees an increasing number of options for health insurance.^2^ Enrollees must choose between plans organized into metal tiers (ie, platinum, gold, silver, bronze, catastrophic) defined by actuarial value (AV),^3^ with varying premiums, deductibles, and cost-sharing. Each year, enrollees are then retrospectively assigned an individual risk score by the Department of Health and Human Services’ (HHS) Hierarchical Condition Category (HCC) risk adjustment model.^4^ These risk scores are used to compensate insurers whose enrollees are coded as higher risk than the statewide average to protect against adverse selection and mitigate insurers’ incentives to attract low- vs high-risk enrollees.^5^

Overall rates of cost sharing for marketplace enrollees have increased over time due to the increased prevalence of low AV plans from insurers.^6^ In theory, low AV plans may address rising national health expenditures by incentivizing more enrollees to become discerning consumers of health care based on price and quality.^7^ The low premiums and high deductibles associated with these offerings may be attractive to individuals who expect to have good health, low utilization of care, or an ability to absorb unexpected medical costs. However, enrollees in low AV plans frequently have higher out-of-pocket (OOP) expenses, especially among those living with chronic conditions.^8,9,10^ Furthermore, these plans may lead to increased medical debt and deferred care.^11^ Low-risk individuals are typically better off with health plans that trade lower monthly premiums for more cost-sharing in the rare instances of substantial medical spending.^12^ In contrast, individuals who are likely to have high costs may rationally choose to trade higher premiums for less cost sharing in higher AV plans. These differences in incentives may lead to differential sorting into metal tiers based on enrollees’ forecasts of their own health risks and needs.^13^

Previous research has identified the aggregate demographic characteristics, health status,^14^ and insurance choices of individual marketplace enrollees.^15^ However, this research does not provide detail on enrollee plan selection and cost implications across varying levels of enrollee risk, which limits understanding of the associations between these factors. Furthermore, studies have shown many enrollees make suboptimal selections, despite decision-making nudges,^16,17,18,19^ due in part to lack of health insurance literacy and variable assessment of their own health risk.^19^ A greater understanding of the distribution of enrollee risk and spending by metal tier may enhance the ability of market administrators, insurance plan designers, and policy makers to offer appropriate insurance products.

Our objectives in this study were to (1) identify differences in marketplace enrollee’s metal tier selections by HHS-HCC risk score; (2) identify differences in total and OOP cost by metal tier, stratified by HHS-HCC risk score; and (3) illustrate the distribution in total and OOP cost among enrollees of varying risk levels. Taken together, our findings may elucidate the complex and previously unknown associations between patient risk, metal tier selection, and health spending within ACA qualified health plans (QHPs). While no analysis can comprehensively explain enrollee decision-making, we hope to address what has been a blind spot in the literature, offer our hypotheses, and propose next steps for future research efforts.

## Methods

### Data Source

We obtained administrative claims data for QHP enrollees in 2019 through a partnership with the Wakely Consulting Group, an actuarial consulting firm. Wakely maintains a nationally representative, deidentified database for ACA QHPs that includes claims data extracted from the Centers for Medicare & Medicaid Services Enrollee-Level Data Gathering Environment server.^20^ These data are routinely collected by insurers, who voluntarily submit their data to Wakely as a part of a national profitability study^21^—insurers who do not agree to submit their data to Wakely are not included in the data set. The database contained more than 4 million unique enrollees in 2019 for the small group, individual, and catastrophic markets. We limited our sample to individual market enrollees aged 18 to 64 years with full-year coverage within the same plan. Partial-year enrollees were excluded as they are adversely selected^22^ and may introduce data inconsistencies due to cost annualization (eFigure 1 in Supplement 1 provides a sample selection flowchart). For all enrollees, we extracted demographic characteristic data, including age at enrollment end date, sex, census area, and dependent status; data on race and ethnicity were not available. We then obtained data on metal tier selection; plan type (eg, health maintenance organization, preferred provider organization); whether the plan was purchased on-exchange vs off-exchange; metal tier; whether the plan was eligible for cost-sharing reductions (CSRs), a subsidy that reduces cost-sharing for low-income enrollees in eligible silver plans; and all associated medical and pharmacy claims. The study was deemed to be exempt by the Vanderbilt University Medical Center Institutional Review Board with a waiver of informed consent due to the impossibility of obtaining consent from the number of enrollees. Our study method adhered to the Strengthening the Reporting of Observational Studies in Epidemiology (STROBE) reporting guideline for cross-sectional studies.^23^

### HHS-HCC Risk Score Primer

The ACA established policies for risk adjustment to provide certainty and protection against adverse selection in individual marketplaces.^24^ An HHS-HCC risk score is calculated for each enrollee based on their age, sex, metal tier, enrollment duration, prescription drug categories, and presence of major comorbidities as categorized by HCCs.^4^ Thus, individuals who have no utilization of medical services still have an HHS-HCC risk score. Mean plan-level scores for all enrollees are used to transfer payments from low- to high-risk plans and mitigate incentives for insurers to selectively attract and enroll low-risk individuals to enhance their profitability or other enrollee selection activity by insurance companies.^25,26,27^ In the HHS-HCC model, risk scores among identical enrollees vary by metal level due to induced demand, unobserved preferences, and ability to access care.

### Statistical Analysis

Data analysis was conducted from March 2021 to January 2023. First, we calculated standardized enrollee risk scores using published platinum coefficients from the HHS-HCC risk adjustment model.^28^ This step allowed us to compare enrollees with equal health risks across metal tiers. Second, we calculated the proportion of members who enrolled in each metal tier by HHS-HCC risk percentile, allowing us to estimate how mean plan selection varied with enrollee risk. Third, we calculated the following cost measures for each enrollee: total spend (synonymous with total allowed amount, the sum of all contracted rates for all submitted claims), total paid (amount disbursed by the insurer, inclusive of CSR contribution), and total OOP costs owed by the enrollee (calculated as total spend minus total paid). We stratified these calculations by risk score percentile, metal tier, and CSR type. We also compared absolute spending differences by metal tier across risk score deciles and calculated risk-normalized average total spend by averaging the total spend by each metal tier across risk deciles. Fourth, we assessed whether the observed differences in spending and enrollment among metal tiers were statistically significant. Since our dependent variable is ordinal (metal tier), we used either χ^2^ tests for outcomes that are counts or proportions or Kruskal-Wallis tests for continuous outcomes. Statistical significance was determined at the α = .05 level; all analyses were conducted via Microsoft R Open, version 4.0.2 (Microsoft Corp).

## Results

### Sample Description

Enrollment and claims data were obtained for 1 317 707 individual market QHP enrollees aged 18 to 64 years (53.5% female; 46.5% male; mean [SD] age, 46.35 [13.43] years), representing all US census areas and sexes (Table). These enrollees were distributed across all age groups, with a larger share of enrollees in older age groups. A total of 34.6% of enrollees were in plans with a CSR. Primary subscribers constituted 74.5% of the sample enrollees vs 25.5% dependents, and 84.0% of enrollees submitted at least 1 claim throughout the year. The HHS-HCC risk score distribution had a long tail—a histogram of risk scores is available in eFigure 2 in Supplement 1. A total of 75.5% of enrollees did not have any HCCs assigned, and thus their risk score was strictly a function of age, sex, and enrollment duration. This is consistent with 2019 Centers for Medicare & Medicaid Services data, which reported that 22.9% of non-catastrophic enrollees have at least 1 assigned HCC. As a result, at the low end of the risk spectrum, some discrete risk scores represented more than 1% of the population. These percentile groups were combined in subsequent analyses of enrollment and cost.

**Table.** Characteristics of the Study Sample^a^

Variable	No. (%)
Age category, y	
18-25	120 176 (9.1)
26-29	85 798 (6.5)
30-39	218 568 (16.6)
40-49	246 093 (18.7)
50-59	371 498 (28.2)
60-64	275 574 (20.9)
Sex	
Female	704 945 (53.5)
Male	612 762 (46.5)
Geographic region	
Northeast	235 363 (17.9)
Midwest	260 880 (19.8)
South	517 100 (39.2)
West	304 364 (23.1)
Submitted any claims	
Yes	1 106 389 (84.0)
No	211 318 (16.0)
Plan type	
Health maintenance organization	434 031 (32.9)
Preferred provider organization	99 084 (7.5)
Exclusive provider organization	351 076 (26.6)
Point of service	13 514 (1.0)
Information unavailable	420 002 (31.9)
Exchange status	
Off-exchange	202 673 (15.4)
On-exchange	1 115 034 (84.6)
Metal level	
Platinum	17 744 (1.3)
Gold	115 011 (8.7)
Silver	635 117 (48.2)
Silver 70%	178 980 (13.6)
Silver 73%	67 105 (5.1)
Silver 87%	179 033 (13.6)
Silver 94%	209 999 (15.9)
Bronze	533 958 (40.5)
Catastrophic	15 877 (1.2)

^a^ Data obtained from the 2019 Wakely Consulting Group Affordable Care Act database.

### Enrollment

**Figure 1.**
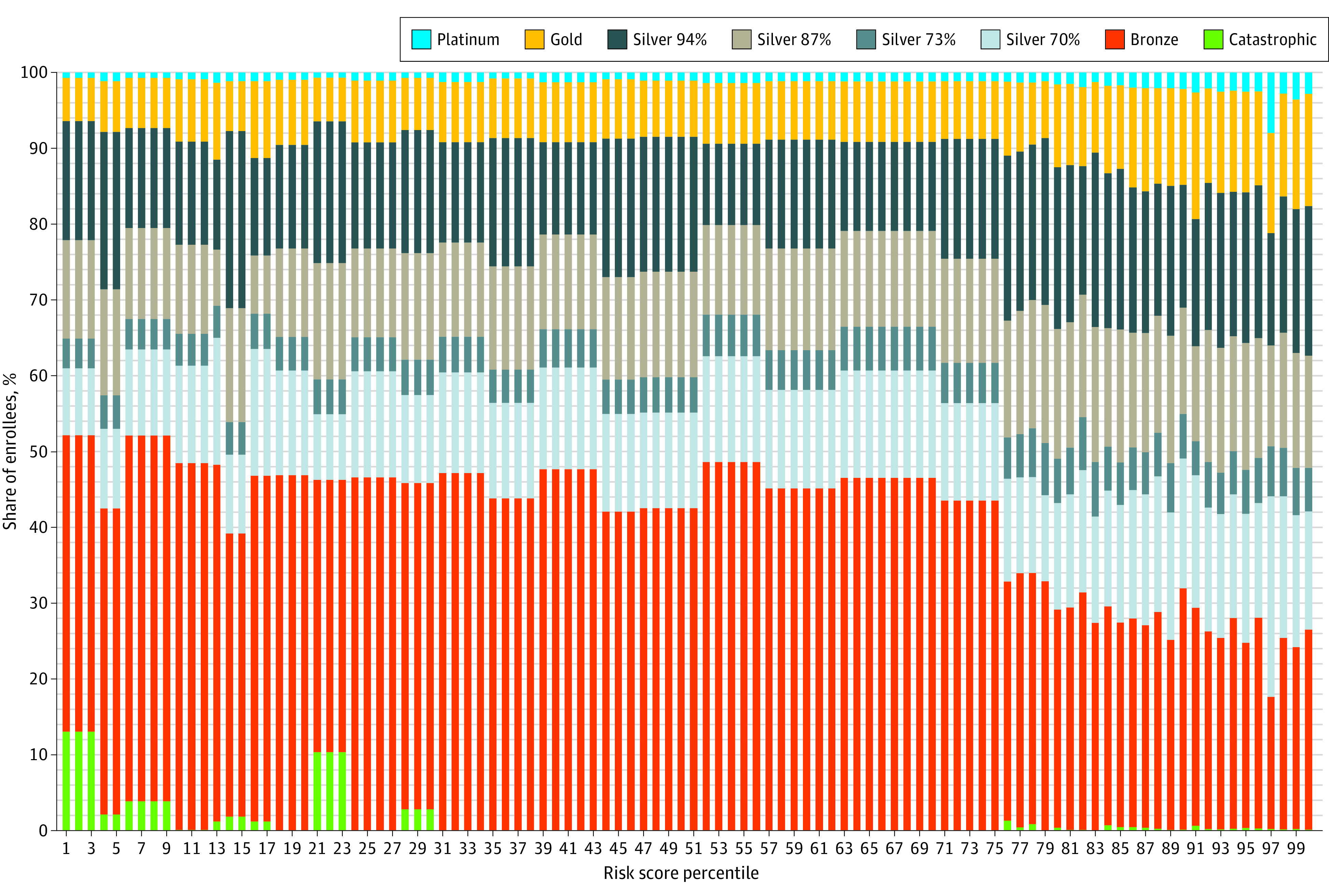
Metal Tier Enrollment by the Department of Health and Human Services (HHS) Risk Score Percentile HHS-Hierarchical Condition Category risk scores for all enrollees were calculated using the coefficients for platinum tier insurance. Data obtained from the 2019 Wakely Consulting Group Affordable Care Act database.

Metal tier selection varied significantly by risk score quartile (χ^2^ = 63 337; *df* = 15; *P* < .001), and these differences appear to be based on the higher-risk enrollees (Figure 1). Platinum (42.0%) and gold (34.4%) plan enrollees were the most likely to be classified as high risk (within the top quartile of risk score), compared with silver CSR (29.7%), silver non-CSR (29.6%), bronze (17.2%), and catastrophic (6.8%). These differences were statistically significant (χ^2^ = 36 102; *df* = 5; *P* < .001). Among the top risk score quartile, CSR plan enrollees constituted a larger share of total enrollees (41.3%) than they did in the bottom 3 risk score quartiles (32.4%).

### Total Spending

**Figure 2.**
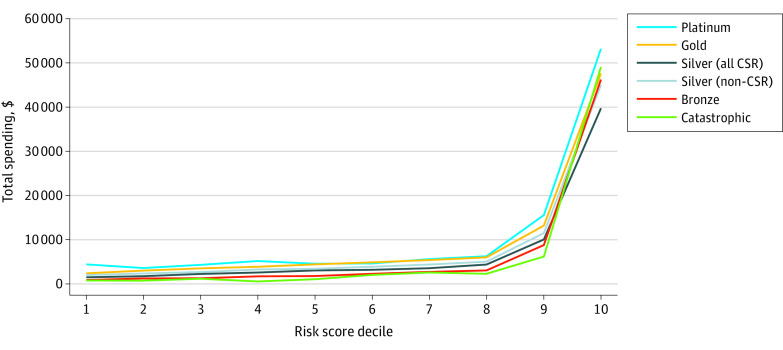
Total Spend Amount by Risk Score Decile and Metal Tier Department of Health and Human Services’ Hierarchical Condition Category risk scores for all enrollees were calculated using the coefficients for platinum tier insurance. Data obtained from the 2019 Wakely Consulting Group Affordable Care Act database. CSR indicates cost-sharing reduction.

**Figure 3.**
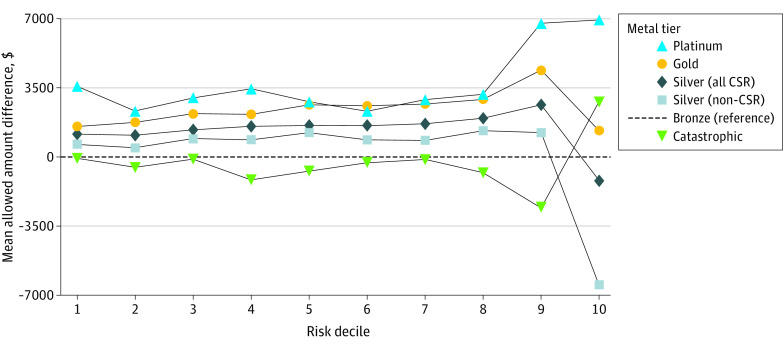
Difference in Mean Total Spend by Metal Tier and Risk Score Decile For each metal tier and decile, absolute difference in total spend was calculated by subtracting the bronze mean total spend from the given metal tier’s spend. Department of Health and Human Services’ Hierarchical Condition Category risk scores for all enrollees were calculated using the coefficients for platinum tier insurance. Data obtained from the 2019 Wakely Consulting Group Affordable Care Act database. CSR indicates cost-sharing reduction.

Median total spend varied significantly by metal tier (Kruskal-Wallis H = 97 070; *df* = 5; *P* < .001): $297 (IQR, $0-$954) for catastrophic, $593 (IQR, $28-$2100) for bronze, $1494 (IQR, $340-$5116) for silver (CSR), $1923 (IQR, $540-$6174) for silver (non-CSR), $2675 (IQR, $728-$9070) for gold, and $4111 (IQR, $728-$9070) for platinum plans. In the bottom 9 deciles, spend was lowest in the bronze metal tier, excluding catastrophic plans, which had few enrollees (Figure 2 and Figure 3). In the top risk score decile, silver CSR plans had the lowest total spend, with a gap greater than 10% from the next closest metal tier (Figure 3; eFigure 3 in Supplement 1). Risk-normalized total spend for the silver 94% AV plan was most comparable with bronze plans (1.2% difference) (eFigure 3 in Supplement 1), while non-CSR plans of similar AV (eg, platinum, 90% AV), had substantially more total spend across risk deciles (Figure 3).

Total spend distribution by HHS-HCC risk score percentile is reported in eFigure 4 in Supplement 1. For enrollees in the bottom 3 quartiles of risk adjustment scores who largely had no HCC comorbidities, 21.3% had $0 total spend and 89.6% had greater than $2000. The proportion of enrollees with $0 total spend also varied by metal tier (χ^2^ = 35 000; *df* = 5; *P* < .001); the highest share was noted in the catastrophic (26.4%) and bronze (22.7%) plans, while gold plans had the lowest share (8.1%) (eFigure 5 in Supplement 1).

### OOP Costs

**Figure 4.**
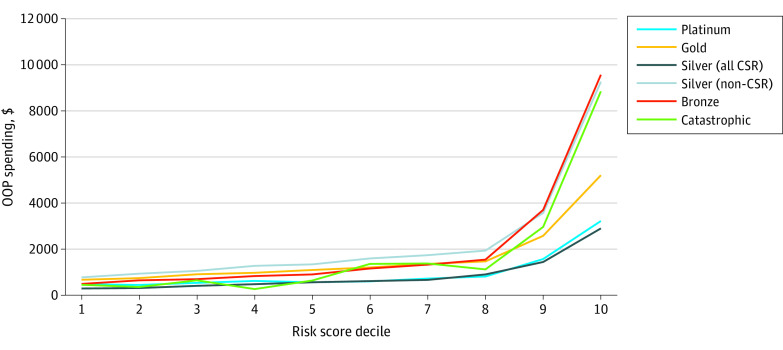
Average Out-of-Pocket (OOP) Cost by Risk Score Decile and Metal Tier Department of Health and Human Services’ Hierarchical Condition Category risk scores for all enrollees were calculated using the coefficients for platinum tier insurance. All silver plans are aggregated; eFigure 6 in Supplement 1 provides a breakdown by CSR type. Data obtained from the 2019 Wakely Consulting Group Affordable Care Act database.

Median OOP cost also varied significantly by metal tier (Kruskal-Wallis H = 56 822; *df* = 5; *P* < .001): $59 for catastrophic, $225 for bronze, $228 for silver (CSR), $724 for silver (non-CSR), $812 for gold, and $498 for platinum plans. Enrollee’s OOP costs by metal tier and risk score decile can be seen in Figure 4. Costs among non-CSR-eligible plans were qualitatively similar for enrollees without HHS-HCC comorbidities (bottom 7 deciles). Enrollees in bronze, catastrophic, and silver 70% AV plans had higher average OOP costs for risk score deciles 8 through 10 (eFigure 6 in Supplement 1).

The distribution of OOP costs by HHS-HCC risk score percentile is shown in eFigure 7 in Supplement 1. Higher risk score quartiles were associated with higher OOP costs (*P* < .001). For enrollees in the bottom 3 quartiles of risk scores, 10.1% had less than $2000 in OOP costs; however, in the top quartile of risk score, 41.0% passed this mark. Additionally, across all percentiles of risk score, there was a small proportion of enrollees who had $0 OOP spend.

## Discussion

The associations between metal tier selection, risk score, and health spending reflect the complexity of health insurance decision-making. Metal tier selection for individual QHP enrollees was associated with HHS-HCC risk score, with larger shares of enrollees in higher AV plans being classified as high risk (within the top risk quartile). However, sizeable shares of enrollees in low AV bronze plans were also labeled high risk (17%). This may indicate inaccurate self-assessment of future health spending or a lack of available alternatives. However, individuals who receive minimal or no subsidies and have substantial and predictable utilization of care may minimize costs (sum of premiums and OOP) with a low-AV plan, particularly if they would reach the maximum OOP limit.

In contrast, a substantial portion of low-risk score enrollees purchased gold or platinum plans, which may indicate higher risk aversion and willingness to protect against unanticipated medical costs,^29^ or reduced effective premiums of gold plans for subsidized individuals due to the effects of “silverloading” on premium subsidies. Silverloading is the strategy where insurers increased only silver plan premiums to compensate for the loss of direct federal reimbursement for CSR subsidies which led to more affordable non-silver plans for subsidized ACA buyer.^30^ Furthermore, selection of plans with higher premiums and lower deductibles may be a rational approach for enrollees with high-risk family members on the same plan or among those experiencing financial liquidity constraints.^31^

The Centers for Medicare & Medicaid Services previously reported that nearly one-fourth of enrollees earning below 250% of the federal poverty level are not enrolled in a CSR plan.^32^ This could be due to an association between worse health status and lower incomes (and thus increased CSR eligibility),^33^ or that enrollees are forward-looking with regard to their expected future health spending. Our sample, which includes both on- and off-exchange enrollees, comprised a smaller share of CSR enrollees (35%) than the nationwide average of on-exchange enrollees (54%).^32^

Across the range of enrollee risk scores, bronze plans were associated with lower total spend than platinum and gold plans. This could be associated with enrollees’ improved assessment of their own risk (eg, adverse selection),^34^ differences in benefit generosity (eg, moral hazard),^35^ deferred care,^7,36,37^ and/or new-onset comorbidities. Prior research has noted that even small amounts of cost-sharing may cause patients to avoid both high- and low-value health services, especially among low-income households.^38,39,40^ Thus, the higher OOP costs observed for high-risk enrollees in low-AV plans may have deleterious consequences for health outcomes.

Analyzing risk-normalized total spend shows that CSR enrollees’ utilization differed from enrollees in plans with similar AV and health risk scores. Enrollees in silver 94% AV plans have the most heavily subsidized insurance with the most favorable enrollee cost-sharing—slightly better than the platinum tier average of 90% AV.^3^ However, accounting for health status, the risk-normalized average total spend among these enrollees was most similar to bronze tier enrollees, with a difference of 1.2%. Additionally, among the top decile of risk score, total cost per CSR enrollee was lower than any of the non-CSR plan enrollee populations by more than 10%. These differences in utilization could be due to 2 groups of factors. First, the insurers that cover silver plan CSR enrollees may be substantially different in their networks, utilization management strategies, and formularies than insurers that predominantly cover non-CSR enrollees. Second, silver CSR enrollees have, by definition, relatively lower income, and may consequently face additional barriers that impede their ability to access care. As an illustrative example, lack of access to transportation may discourage low-income patients from pursuing some procedures, even if they face little to no OOP expenses.^41^

More research is needed to evaluate and explain the discrepancy in total spend among high-risk CSR enrollees, as it could represent a disparity in health equity. One potential approach would be to define cohorts of risk-score-matched, comorbidity-matched enrollees with and without CSRs and then analyze the typical cost breakdown among categories (eg, outpatient care, hospitalizations, elective procedures, prescription drugs). Another approach could be to look at one specific service (eg, kidney transplantation for patients with kidney failure), calculate the share of enrollees who receive the service compared with those eligible, and compare between insurer populations (eg, CSR, Medicaid, gold tier).

### Limitations

There are several limitations to our study that should be considered when assessing the generalizability of our findings. First, we were unable to observe premium subsidies, which precluded estimation of net spending by enrollee. Second, demographic information on enrollees was limited and did not contain information on income, race and ethnicity, CSR eligibility (for enrollees in non-CSR plans), or geography smaller than census region. Lack of geographic granularity limits visibility into specific insurance markets and extent of plan selection. Third, we only observed 1 year of data and thus could identify neither an enrollee’s historical risk score and spending nor comorbidities present at enrollment. This necessitated the use of a concurrent risk score in which enrollee health risk is calculated in the same period as spending and utilization. Furthermore, it precluded analysis of adverse selection based on a prior year risk score. Fourth, we were unable to determine the appropriate level of spending from these data and whether certain types of enrollees or plans were more likely to experience unmet care needs due to cost. Fifth, the Wakely Consulting Group ACA database is biased toward risk-adjustment payers, including an average transfer of –$31.25 per-member per-month for individual market QHPs. Sixth, the HHS-HCC risk score is calculated from the combination of observed demographic administrative records, pharmacy use, and clinician-entered diagnosis codes. We were not able to assess the accuracy of the risk score relative to the latent construct of health risk, because coding patterns are not uniform and variation exists as shown in other settings.^42^ Seventh, due to data limitations, we assumed the patient pays all OOP costs; these calculations do not account for bad debt, charity care, indemnity and other secondary insurances, or third-party payments that are unobserved.^43^

## Conclusions

In this cross-sectional study of ACA marketplace enrollees, our findings suggest that enrollee metal tier selection is partially responsive to health risk measured by HHS-HCC risk score and that CSR plan enrollees tend to have lower total spend at the high end of the risk spectrum. It also underscores the need for research on enrollee experiences, enrollee and plan factors that affect metal tier selection, access to care, and selective enrollment to identify opportunities to improve health care decision-making and value in health.

## Notes

Supplement 1. **eFigure 1.** Exclusion Criteria Flowchart **eFigure 2.** Frequency Distribution of Enrollees by Risk Score **eFigure 3.** Total Spend by Metal Tier, Risk Score Decile **eFigure 4.** Distribution of Total Spend by Enrollee Risk Score Percentile **eFigure 5.** Share of Enrollees With $0 in Total Spend **eFigure 6.** Average Total Out-of-Pocket Spending by Metal Tier, Risk Score Docile **eFigure 7.** Out-of-Pocket Cost Distribution of Enrollees by Risk Score Percentile

Supplement 2.
Data Sharing Statement
