# Supplementary material for: Plan Selection, Enrollee Risk, and Health Spending on the Patient Protection and Affordable Care Act Individual Marketplaces, 2019
Source: JAMA Netw Open. 2023 Mar 30;6(3):e234529. doi: 10.1001/jamanetworkopen.2023.4529 (PMC10064254; doi:10.1001/jamanetworkopen.2023.4529)
Supplement: Supplement 1. — eFigure 1. Exclusion Criteria Flowchart eFigure 2. Frequency Distribution of Enrollees by Risk Score eFigure 3. Total Spend by Metal Tier, Risk Score Decile eFigure 4. Distribution of Total Spend by Enrollee Risk Score Percentile eFigure 5. Share of Enrollees With $0 in Total Spend eFigure 6. Average Total Out-of-Pocket Spending by Metal Tier, Risk Score Docile eFigure 7. Out-of-Pocket Cost Distribution of Enrollees by Risk Score Percentile [file jamanetwopen-e234529-s001.pdf]

Supplementary Online Content

Treasure G, Anderson DM, Hatcher L, et al. Plan selection, enrollee risk, and health spending on the Patient Protection and Affordable Care Act individual marketplaces, 2019. *JAMA Netw Open*. 2023;6(3):e234529. doi:10.1001/jamanetworkopen.2023.4529

eFigure 1. Exclusion Criteria Flowchart

eFigure 2. Frequency Distribution of Enrollees by Risk Score

eFigure 3. Total Spend by Metal Tier, Risk Score Decile

eFigure 4. Distribution of Total Spend by Enrollee Risk Score Percentile

eFigure 5. Share of Enrollees With $0 in Total Spend

eFigure 6. Average Total Out-of-Pocket Spending by Metal Tier, Risk Score Docile

eFigure 7. Out-of-Pocket Cost Distribution of Enrollees by Risk Score Percentile

This supplementary material has been provided by the authors to give readers additional information about their work.

© 2023 Treasure G et al. *JAMA Network Open.*

eFigure 1. Exclusion criteria flowchart

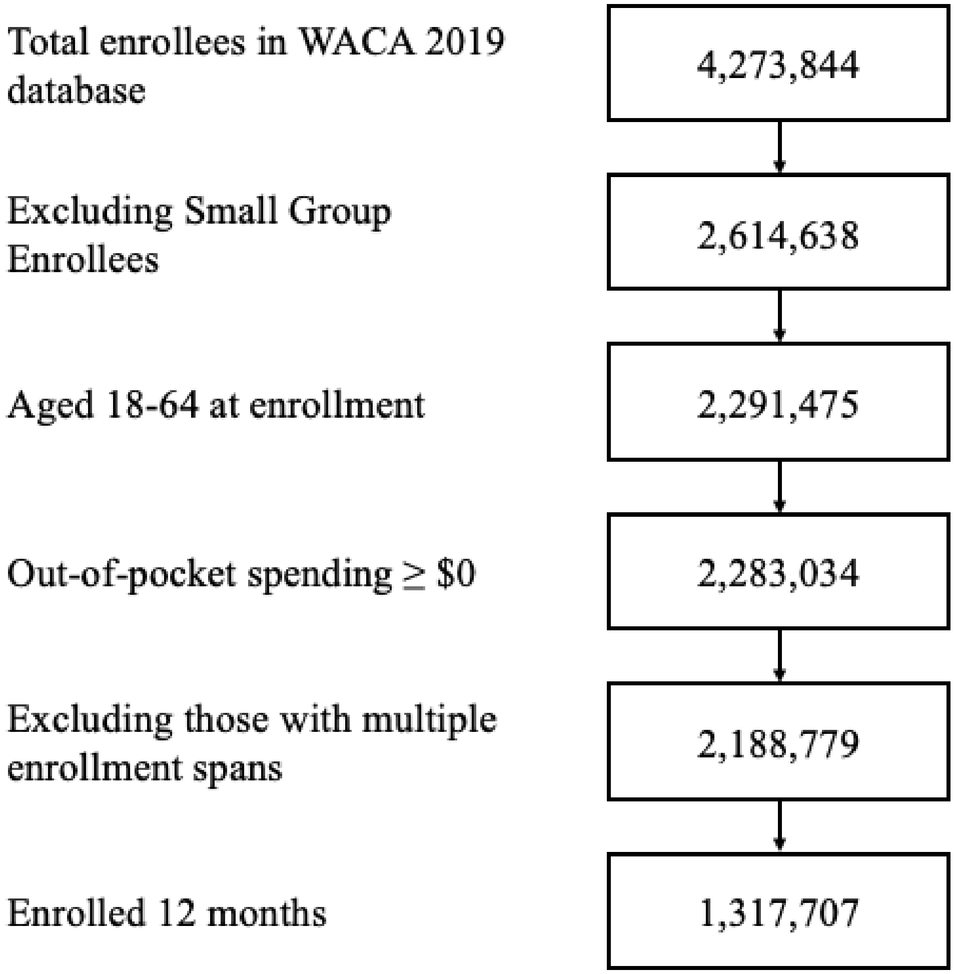

Source: Authors' analysis of data from the 2019 Wakely ACA Database (WACA).

© 2023 Treasure G et al. *JAMA Network Open.*

eFigure 2: Frequency distribution of enrollees by risk score

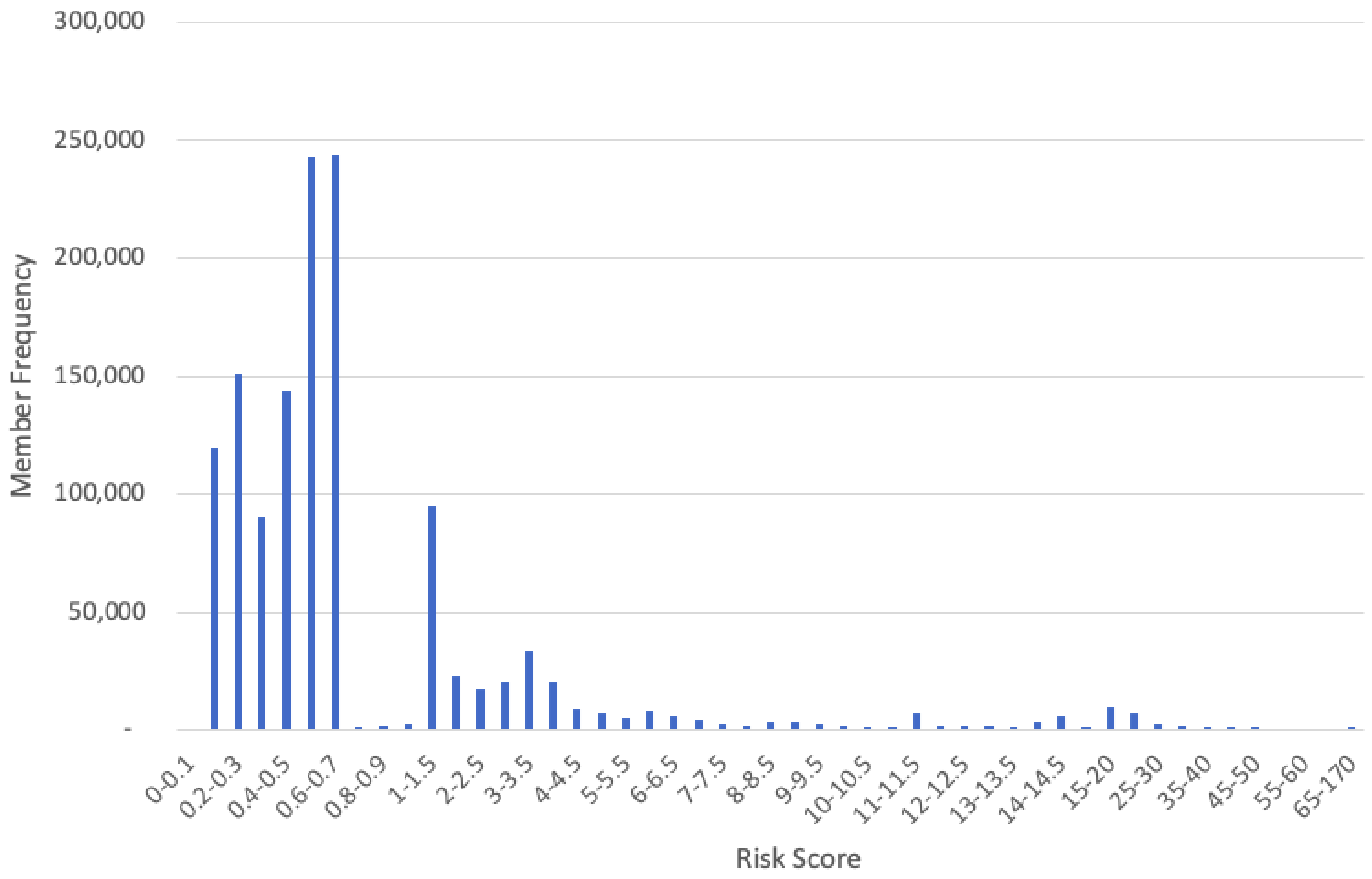

Source: Authors' analysis of data from the 2019 Wakely ACA Database (WACA). **Notes:** The spike at the 1-1.5 range corresponds to risk scores that are calculated with demographics plus more mild comorbidities. Risk adjustment scores that are lower than this are driven almost entirely by demographics alone.

© 2023 Treasure G et al. *JAMA Network Open.*

eFigure 3: Total Spend by Metal Tier, Risk Score Decile *($)*

Decile	Gold	Platinum	Silver (all CSR)	Silver (non-CSR)	Bronze	Catastrophic	Silver 73%	Silver 87%	Silver 94%
**1**	2,382	4,390	1,464	1,983	834	747	1,393	1,560	1,406
**2**	2,972	3,536	1,693	2,323	1,224	704	1,502	1,808	1,656
**3**	3,455	4,259	2,208	2,648	1,269	1,143	1,854	2,394	2,162
**4**	3,834	5,119	2,540	3,230	1,673	512	2,268	2,674	2,511
**5**	4,401	4,547	2,987	3,357	1,760	1,036	2,696	3,136	2,953
**6**	4,852	4,576	3,130	3,844	2,269	1,974	2,681	3,287	3,140
**7**	5,353	5,573	3,509	4,350	2,673	2,544	3,240	3,705	3,429
**8**	5,949	6,188	4,361	4,987	3,030	2,251	3,921	4,531	4,356
**9**	13,167	15,522	10,001	11,417	8,767	6,178	8,927	10,331	10,063
**10**	47,462	53,049	39,613	44,923	46,120	48,943	37,123	41,503	38,798
**Risk-Normalized Mean Spend,** *Difference from Bronze (%)*	34.8%	53.3%	2.7%	19.3%	0% (Reference)	-5.2%	-5.8%	7.6%	1.2%

Source: Authors' analysis of data from the 2019 Wakely ACA Database (WACA). **Notes:** Silver Cost-Savings Reduction (CSR) plan total spend was calculated by taking the average of Silver 73%, Silver 87%, and Silver 94% plans, weighted by total number of enrollees in each plan type. Risk-Normalized Mean Cost Difference was calculated as the average cost across all 10 risk deciles, and compared to the Bronze plan.

© 2023 Treasure G et al. *JAMA Network Open.*

eFigure 4: Distribution of total spend by enrollee risk score percentile

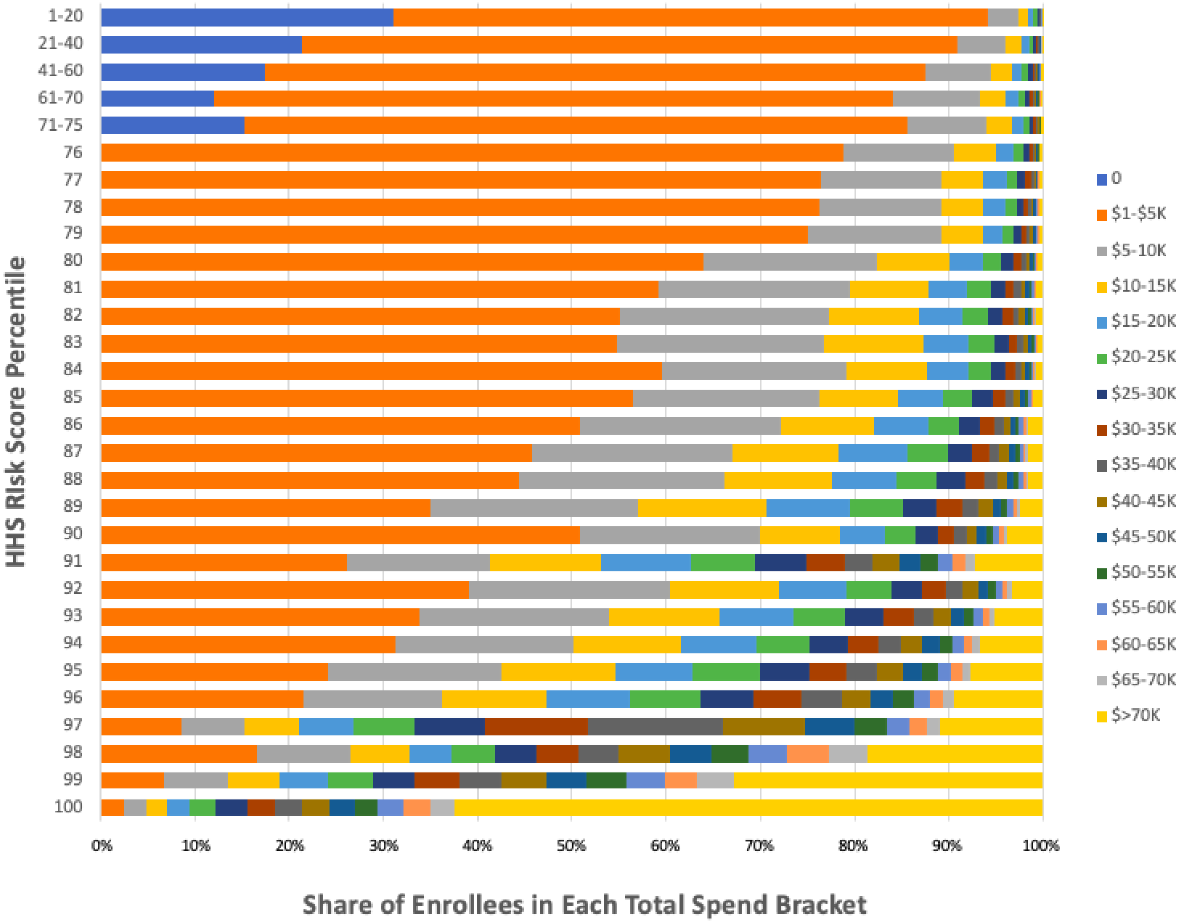

Source: Authors' analysis of data from the 2019 Wakely ACA Database (WACA). **Notes:** HHS-HCC risk scores for all enrollees were calculated using the coefficients for platinum tier insurance. At the lower end of the risk distribution, scores were close together, varying by age and gender, and not by comorbidities. Thus, enrollees at the lower end of the risk distribution were grouped. ***FOR REVIEW ONLY:*** We are aware that the distribution in the 91st percentile is different from the surrounding percentiles, we have verified that this is by chance and not a data error.

© 2023 Treasure G et al. *JAMA Network Open.*

eFigure 5: Share of enrollees with $0 in total spend

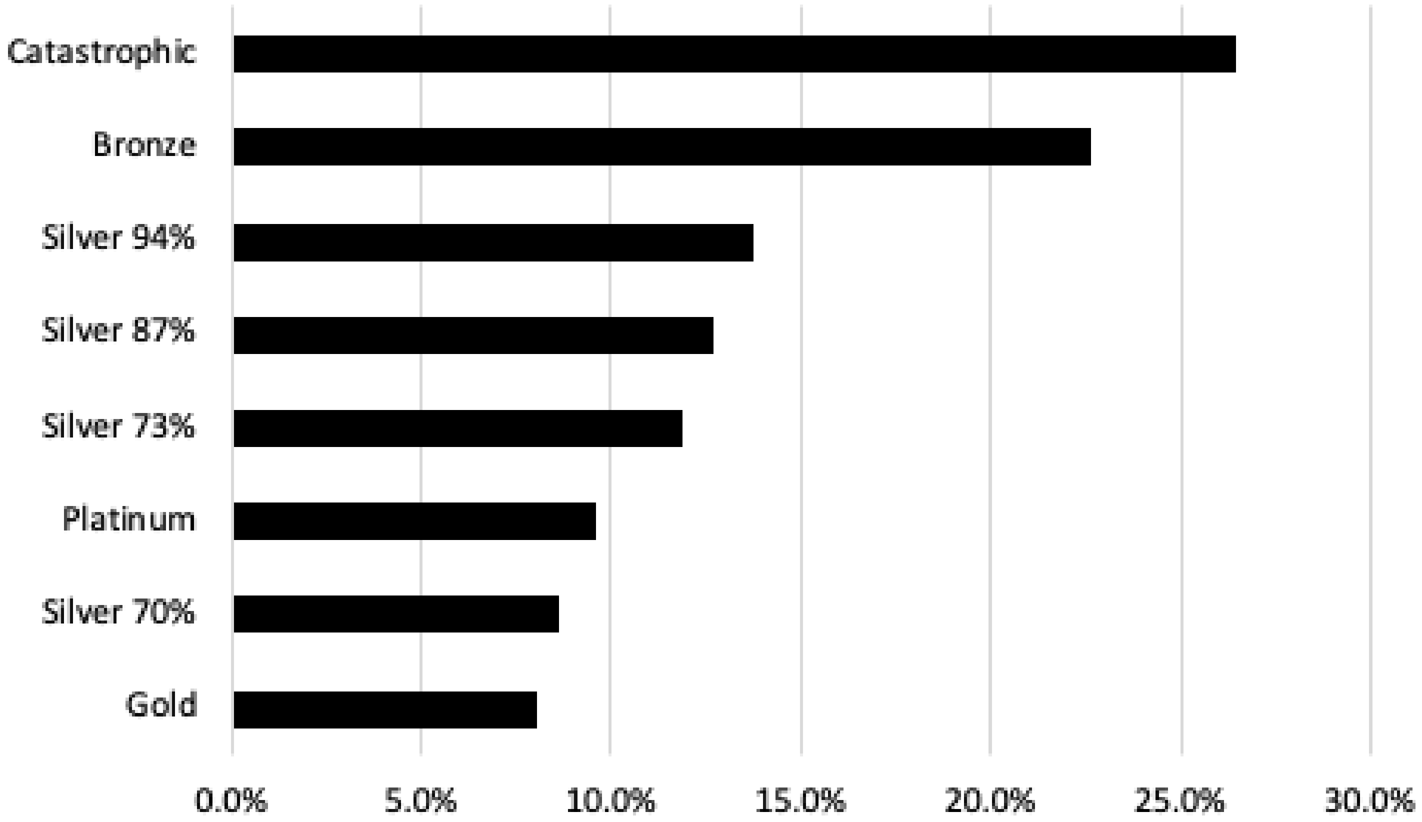

Source: Authors' analysis of data from the 2019 Wakely ACA Database (WACA).

© 2023 Treasure G et al. *JAMA Network Open.*

eFigure 6: Average Total Out-of-Pocket Spending by Metal Tier, Risk Score Decile *($)*

Decile	Gold	Platinum	Silver (all CSR)	Silver (non-CSR)	Bronze	Catastrophic	Silver 70%	Silver 73%	Silver 87%	Silver 94%
**1**	665	477	286	768	484	449	768	530	325	174
**2**	731	443	303	926	646	351	926	579	341	182
**3**	905	531	403	1,050	686	645	1,050	700	473	248
**4**	965	618	473	1,267	831	262	1,267	958	498	295
**5**	1,093	554	561	1,334	893	630	1,334	1,176	580	348
**6**	1,203	597	605	1,594	1,155	1,347	1,594	1,033	689	396
**7**	1,346	714	664	1,727	1,318	1,370	1,727	1,218	753	412
**8**	1,459	807	891	1,921	1,532	1,120	1,921	1,513	958	635
**9**	2,567	1,567	1,435	3,563	3,697	2,960	3,563	2,660	1,549	947
**10**	5,192	3,215	2,889	9,252	9,552	8,833	9,252	5,451	3,126	1,869

Source: Authors' analysis of data from the 2019 Wakely ACA Database (WACA).

© 2023 Treasure G et al. *JAMA Network Open.*

eFigure 7: Out-of-pocket cost distribution of enrollees by risk score percentile

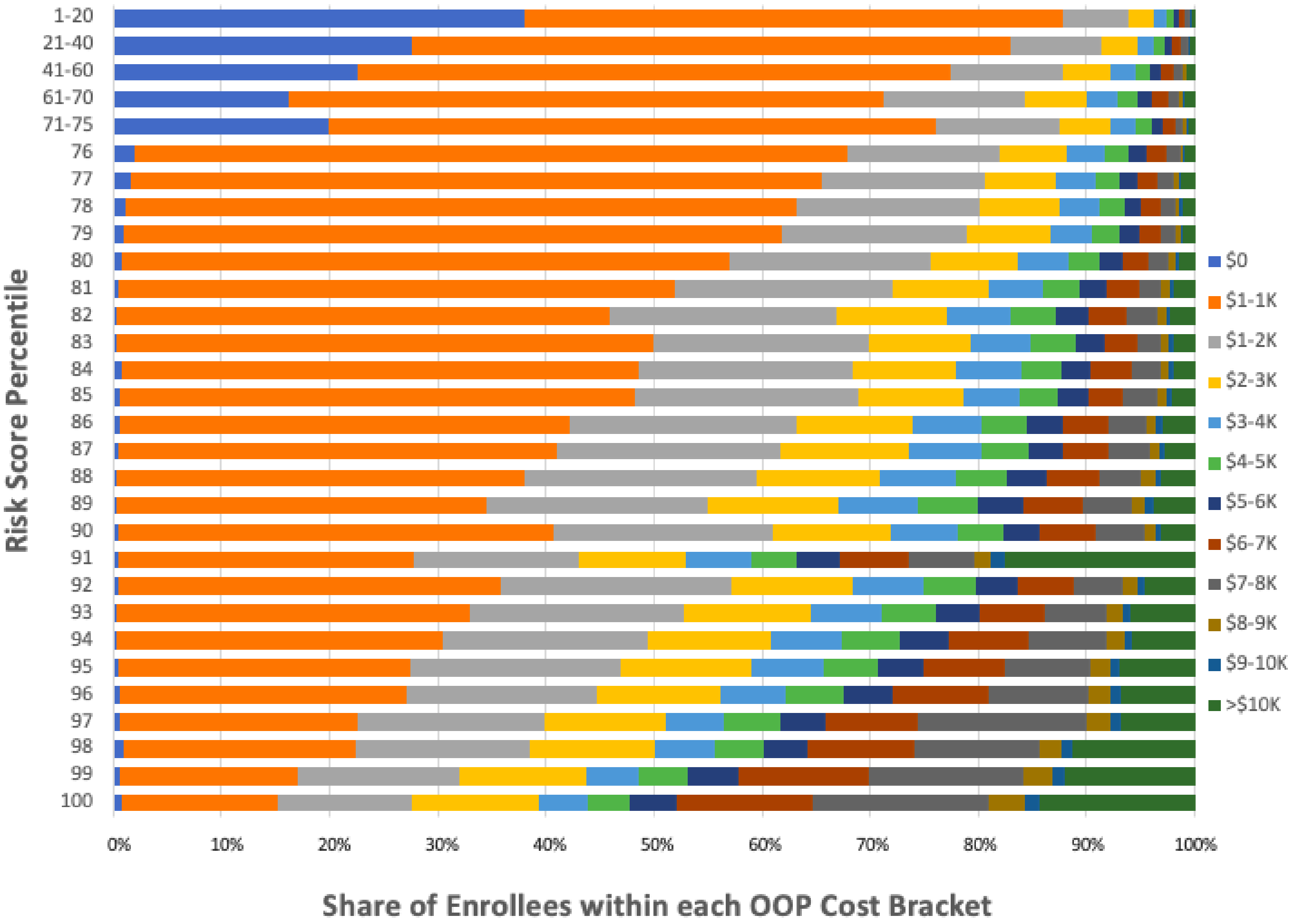

Source: Authors' analysis of data from the 2019 Wakely ACA Database (WACA). **Notes:** Out-of-pocket (OOP) costs were calculated by determining total spend (sum of all eligible claims) minus total paid claims. The OOP calculation includes both deductible and claims submitted that were later found to be out-of-network. Thus, calculated OOP may exceed a plan's maximum OOP. ***FOR REVIEW ONLY:*** We are aware that the distribution in the 91st percentile is different from the surrounding percentiles, we have verified that this is not a data error.

© 2023 Treasure G et al. *JAMA Network Open.*
