# Supplementary material for: Plan Selection, Enrollee Risk, and Health Spending on the Patient Protection and Affordable Care Act Individual Marketplaces, 2019
Source: JAMA Netw Open. 2023 Mar 30;6(3):e234529. doi: 10.1001/jamanetworkopen.2023.4529 (PMC10064254; doi:10.1001/jamanetworkopen.2023.4529)
Supplement: Supplement 2. — Data Sharing Statement [file jamanetwopen-e234529-s002.pdf]

Data Sharing Statement

Treasure. Plan Selection, Enrollee Risk, and Health Spending on the Patient Protection and Affordable Care Act Individual Marketplaces, 2019. *JAMA Netw Open*. Published March 30, 2023. doi:10.1001/jamanetworkopen.2023.4529

Data
Data available: No

Additional Information
Explanation for why data not available: WACA data require a fee and signed data use agreement
